# Synthesis of Boron Nanosheets in Copper Medium

**DOI:** 10.1038/s41598-019-53851-6

**Published:** 2019-11-22

**Authors:** Shuo Zhao, Yuying Wu, Bo Zhou, Xiangfa Liu

**Affiliations:** 1 Key Laboratory for Liquid−Solid Structural Evolution & Processing of Materials, Ministry of Education, Shandong University, Jinan, 250061 China; 2 Institute of Microstructure and Properties of Advanced Materials, Beijing University of Technology, Beijing, 100124 China

**Keywords:** Synthesis and processing, Two-dimensional materials

## Abstract

Boron has a tendency to form bulk structures due to its unique electron-deficient property, so it’s hard for boron to form sheets in large quantities. Here, we report a novel method for the preparation of boron nanosheets in large quantities by copper medium. The method mainly includes mechanical exfoliation, recombination and extraction. A large number of boron nanosheets with a height of below 6 nm have been prepared in this work. X-ray photoelectron spectroscopy and Raman spectroscopy results confirmed that the nanosheets possess the characteristics of α-rhombohedra boron and β-rhombohedra boron with a high content of boron. Hexagonal and rhombic sheets have been observed and two different growth processes are revealed successfully, which are also the basic structures of boron nanosheets. An interesting phenomenon also have been discovered that high density nanotwins exist in β-Rhombohedra boron sheets and it might stimulate more interest in growth of nanomaterials.

## Introduction

Unlike boron nanotubes^1^, nanospheres^2^ and other boron-based nanomaterials, the preparation of boron nanosheets has always been a difficult problem derived from electron-deficient, highly delocalized covalent bonds^3^. Since the first successful synthesis of single atomic borophene on Ag(111), the interest on boron nanosheets has been inspired once again^4,5^. At the same time, the unique physical and electrical properties have been demonstrated in borophene, such as metallic characteristics^6,7^ and anisotropy of electron transport^4^. The 2D anisotropic Dirac cones in the χ_3_ borophene have been observed successfully, which pave the way for boron sheets to be a superconductor^8^. As a necessary development, Qing Zhong *et al*. synthesized two novel metastable phases of 2D boron sheets, which have been proven to be metallic experimentally^9^. Meanwhile, the characterization of borophere is progressing. For example, the Raman spectroscopy of boron nanosheets have been studied, in which β_12_ and χ_3_ borophene phases are defined, providing a basic experimental understanding on the vibrational properties of boron nanosheets^10^. Recently, the preparation of a honeycomb, graphene-like borophene was reported by using Al(111) surface as substrate^11^. Longjuan Kong *et al*. reviewed the progress that has been made on borophene in terms of synthetic techniques, characterizations and the atomic models and indicated that borophene was just in infancy^3^. There is no doubt that the step of research on boron 2D materials has never stopped, both theoretically and experimentally^12^. In summary, the synthesis of boron nanosheets with high cost and harsh conditions, including ultrahigh vacuum, substrate and pure precursor, is still complex^4,5,11–13^. Very recently, In order to solve the problem that the size of boron nanosheets is too small to be applied in some devices, Rongting Wu *et al*. replaced the substrate with Cu(111) surface, by which large-area single-crystal sheets of borophene were obtained successfully^13^.

Boron is the fifth element in periodic table and three outer shell electrons give it unique electrical properties. Therefore, up to 16 bulk allotropies of boron have been observed or predicted due to the highly delocalized bonds, wherein electron pairs are shared among three atoms or more^14^. In other words, three(or more)-center, two-electron(3c-2e or more) bonds placed in boron make it more likely to form bulk rather than sheet. In brief, It’s difficult for boron to form sheet structures like graphene^4^. In fact, not only boron nanosheet has a great potential to be applied in some electronic devices due to its one-of-a-kind physical and chemical properties, a fairly large specific surface area provides it a broader stage in various fields. For instance, ^10^B, an isotope of boron, possesses an excellent neutron absorption property, so boron nanosheets can be used in targeted drugs for cancer treatment. In a recent study, Xiaoyuan Ji *et al*. reported a top-down synthesis of 2D boron nanosheets for multimodal imaging-guided cancer therapy, suggesting that boron nanosheet has a broad prospect in medical research as well^15^. It’s also worth mentioning that the preparation of boron nanosheets in large quantities could be achieved by coupling thermal oxidation etching and liquid exfoliation technologies in this study. Since then, the mass preparation and application of boron nanosheets have been investigated endlessly. Hongling Li *et al*. reported a boron sheets based supercapacitor by sonication-assisted liquid-phase exfoliation, exhibiting impressive electrochemical performance, excellent energy density and cycling stability^16^. Besides, boron nanosheet is also proposed as an elemental two-dimensional material to effectively catalyze the N_2_ reduction reaction toward NH_3_ synthesis with excellent selectivity^17,18^. Therefore, it has become a reality to apply boron sheets in high performance devices. Here, instead of liquid exfoliation or chemical exfoliation, a novel method was applied to prepare boron nanosheets in large quantities, coupling with mechanical exfoliation, growth in solid copper and extraction.

Previously, high purity boron nanomaterials have been prepared by a reducing reaction in copper melt^19^. As we reported before, both boron nanotubes^20^ and boron spheres^2^ have been obtained via copper medium by controlling the cooling rate and boron content, thus synthesis of boron nanosheets by using copper as an ideal carrier has been proposed in this work. In order to decrease the initial size of boron and facilitate grinding, boron/copper alloys were atomized into powders, through which copper matrix composite strengthened by nanoscale boron has been prepared^21^. Here, a large number of boron nanosheets crystallized in copper by solid diffusion, coupling with a high temperature in a vacuum hot pressure sintering furnace. The twins observed in nanosheet suggest that it may have higher mechanical properties and more special electrical properties. Hopefully, This method of mass preparation may lead to more extensive applications of boron nanosheets.

## Results and Discussions

**Figure 1:**
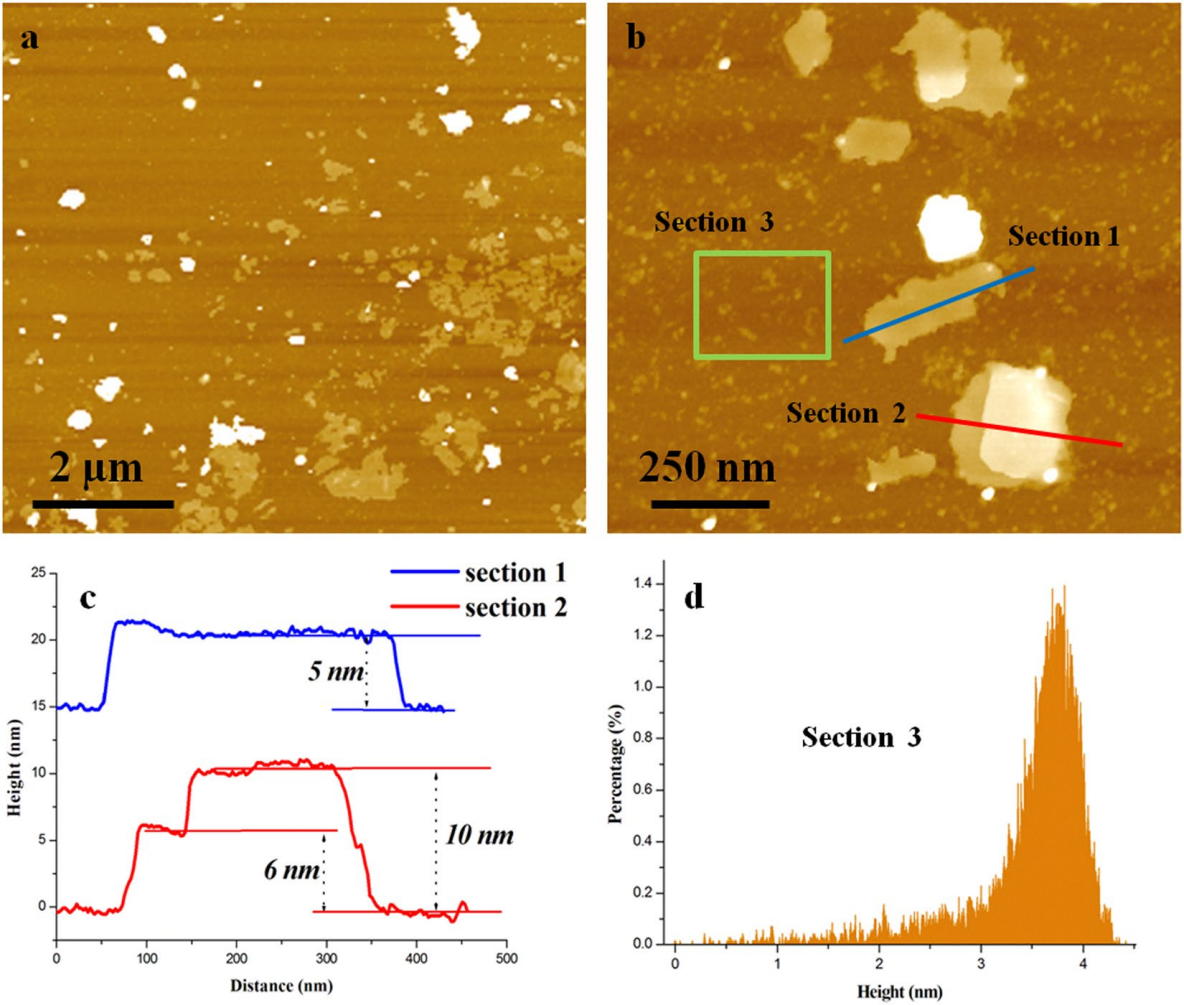
AFM images of boron nanosheets. (**a**) A large number of sheets are shown in the field; (**b**) characterization of specific boron nanosheets by AFM, nanosheets with a less size are also shown in section 3; (**c**) the height-distance curves of two sections labeled in figure (**b**), indicationg that the heights of nanosheets are about 5–6 nm with a lateral size of several hundred nanometers; (**d**) The height statistics of peaks in section(3) and the thickness of most sheets ranges from 2–4 nanometers.

Atomic force microscopy (AFM) equipped on SPM was utilized to characterize the morphology of boron nanosheets. The powders extracted from copper medium were dispersed in alcohol, and then dripped onto a clean, smooth silicon wafer using a straw carefully so as to observation. A large quantity of sheets distributing in the field of vision are shown in Fig. 1(a). It is obvious that the lateral sizes of the sheets range from several nanometers to microns. Section 1 and section 2 in Fig. 1(b) are labeled by blue and red solid lines, representing a single crystalline sheet and overlapping sheets, respectively. Figure 1(c) shows the height distribution of these two sections clearly, indicating that the thickness of the single sheet is about 5–6 nm and that of the overlap is 10 nm, with the lateral size measured several hundred nanometers. In addition to sheets with a relative large area above, many small-scale sheets, the sizes of which are below 5 nm, have also been observed and labeled by green box in Fig. 1(b). The heights of all the peaks are counted in this region and the result has been shown in Fig. 1(d), in which the thickness of most sheets ranges from 2–4 nanometers. It is speculated that the lateral growth is much faster than thickness. As a result, these powders, having large dimensions and relatively small height, could be treated as nanosheets.

**Figure 2:**
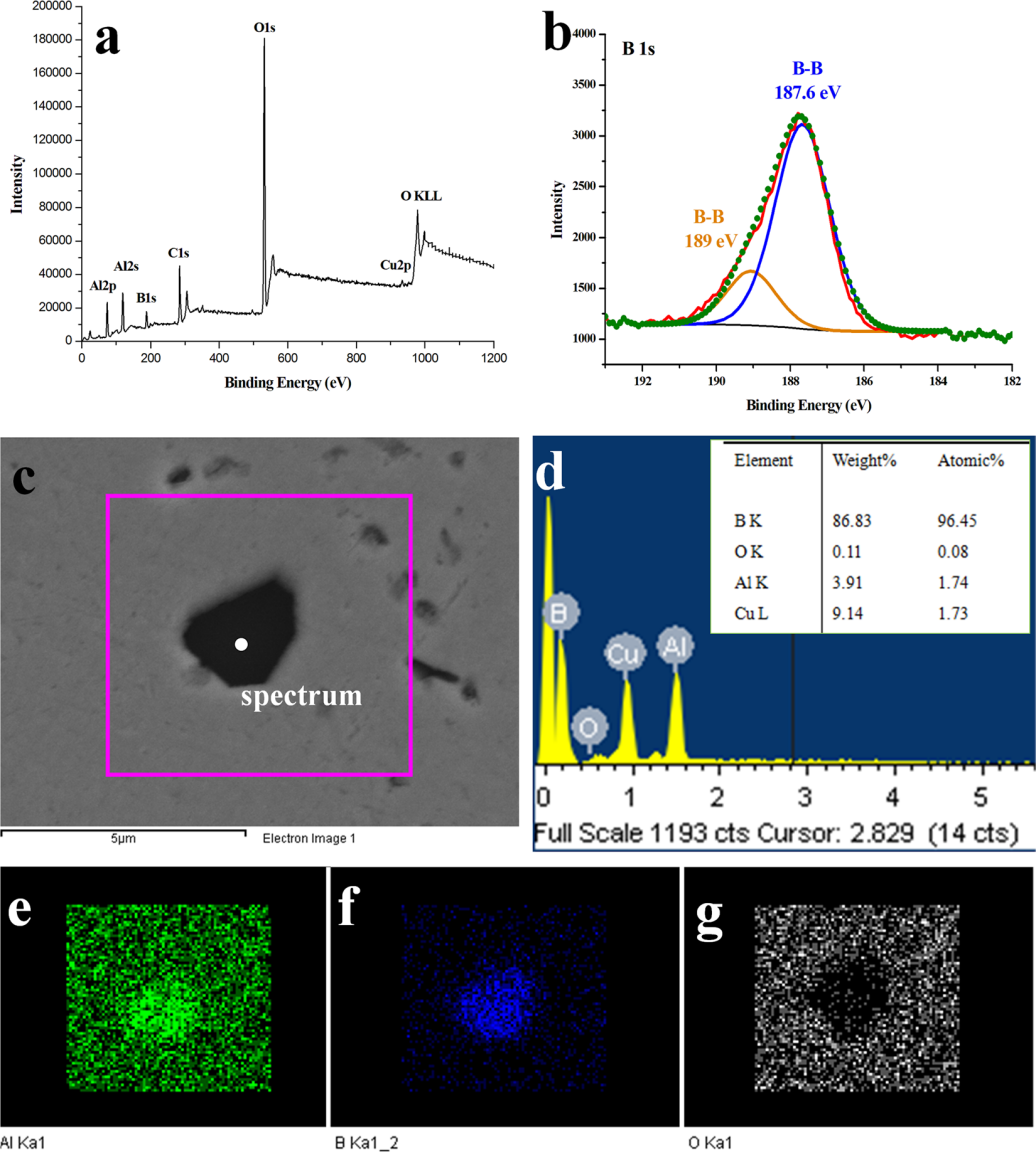
The composition of boron nanosheets. (**a**,**b**) XPS results of the boron nanosheets. Al, O and Cu are dissolved in boron sheets, but (**b**) exhibits pure B-B peaks, in which olive circles represent the data after fitted by blue and orange curves; (**c**–**g**) EDS results of the boron phase in heated Cu-B powders without grinding. More than 96 at% boron is obtained in this process, providing the circumstantial evidence of composition of the nanosheets. Elemental Al tends to dissolve in boron without oxidation, concluded from (**e**–**g**).

In order to investigate the chemical composition and structure of these boron nanosheets, X-rays photoelectron spectroscopy and Raman spectroscopy have been applied. Figure 2(a,b) show the XPS results of the obtained boron nanosheets. Although elemental Al, Cu and O are dissolved in the nanosheets through the wide spectrum, Fig. 2(b) shows pure B-B peaks located at 187.6 eV and 189 eV, which are marked by blue and orange curves respectively, because the 1 s binding energy of bulk boron is located at about 189 eV and that of boron powders will shift to 187 eV. There is no peak observed greater than 190 eV, indicating that the boron nanosheets were hardly oxidized. However it’s hard to identify the composition of boron nanosheets directly due to the small size, so as to further study the specific composition of boron conveniently, raw Cu-B powders without grinding were heated at 1000 °C under a pressure of 30 MPa and the EDS results are exhibited in Fig. 2(c–g). The boron content reaches up to more than 96 at% as shown in the insert image of Fig. 2(d), thus it can be inferred that the nanosheets obtained in milled Cu-B alloys could be regarded as boron nanosheets. Elemental Al, B and O were labeled by green, blue and white in EDS mapping results. An interesting phenomenon has been observed that aluminum tends to dissolve in boron rather than copper through solid diffusion, while very little oxygen was detected, so that aluminum exists only in a dissolved state in boron nanosheets, rather than in an oxidation state.

**Figure 3:**
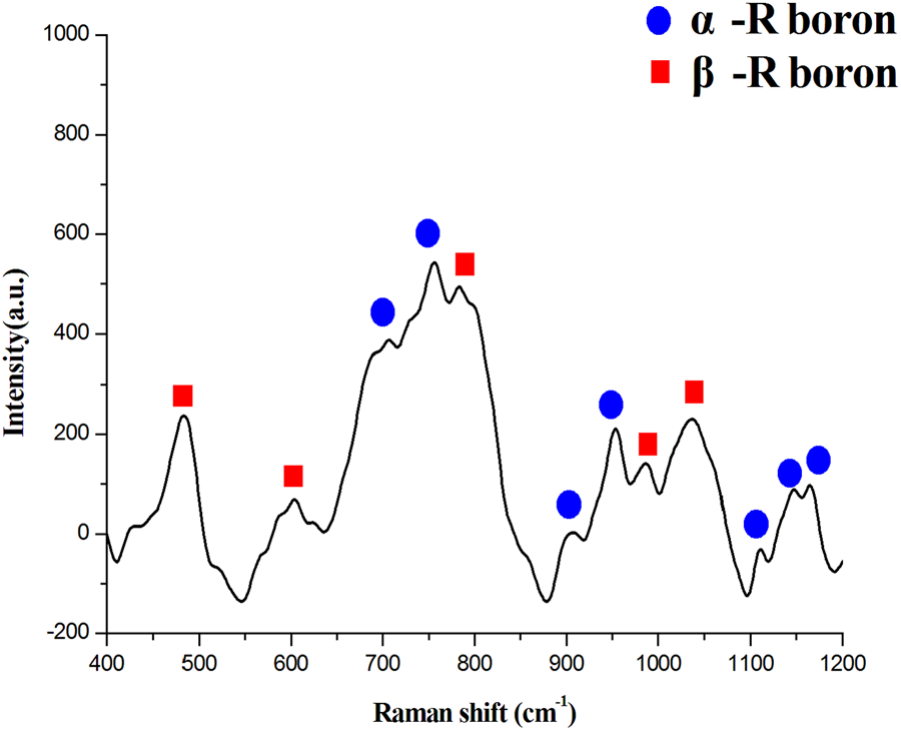
The Raman result of the boron nanosheets, revealing two crystal structures of boron, α-rhombohedra and β-rhombohedra boron, the peaks of which are labeled by blue and red, respectively.

Raman spectroscopy was utilized to identify the crystal structures of boron nanosheets as shown in Fig. 3. According to Parakhonskiy’s investigations, the Raman shift values greater than 1000 cm^−1^ represent inter-icosahedron bonds and that less than 1000 cm^−1^ correspond to intra-icosahedron bonds^22^. Since the basic unit of boron is icosahedron, the bonding between icosahedrons is vital to judge the boron structure, for α-rhombohedra boron especially. The three peaks located at 1118 cm^−1^, 1153 cm^−1^ and 1170 cm^−1^ are typical Raman shift values of alpha rhombohedra boron^23^. In addition, beta rhombohedra boron can also be characterized by Raman spectroscopy due to its unique B_28_ clusters as labeled by red rectangles^24^. This result is basically consistent with our previous report investigating the boron structures in Cu-B alloys^25^, in which eutectic boron had the typical traits of α-B and primary boron was identified as β-B. Therefore, these two structures of boron, α-rhombohedra boron and β-rhombohedra boron, have been determined by Raman spectroscopy.

**Figure 4:**
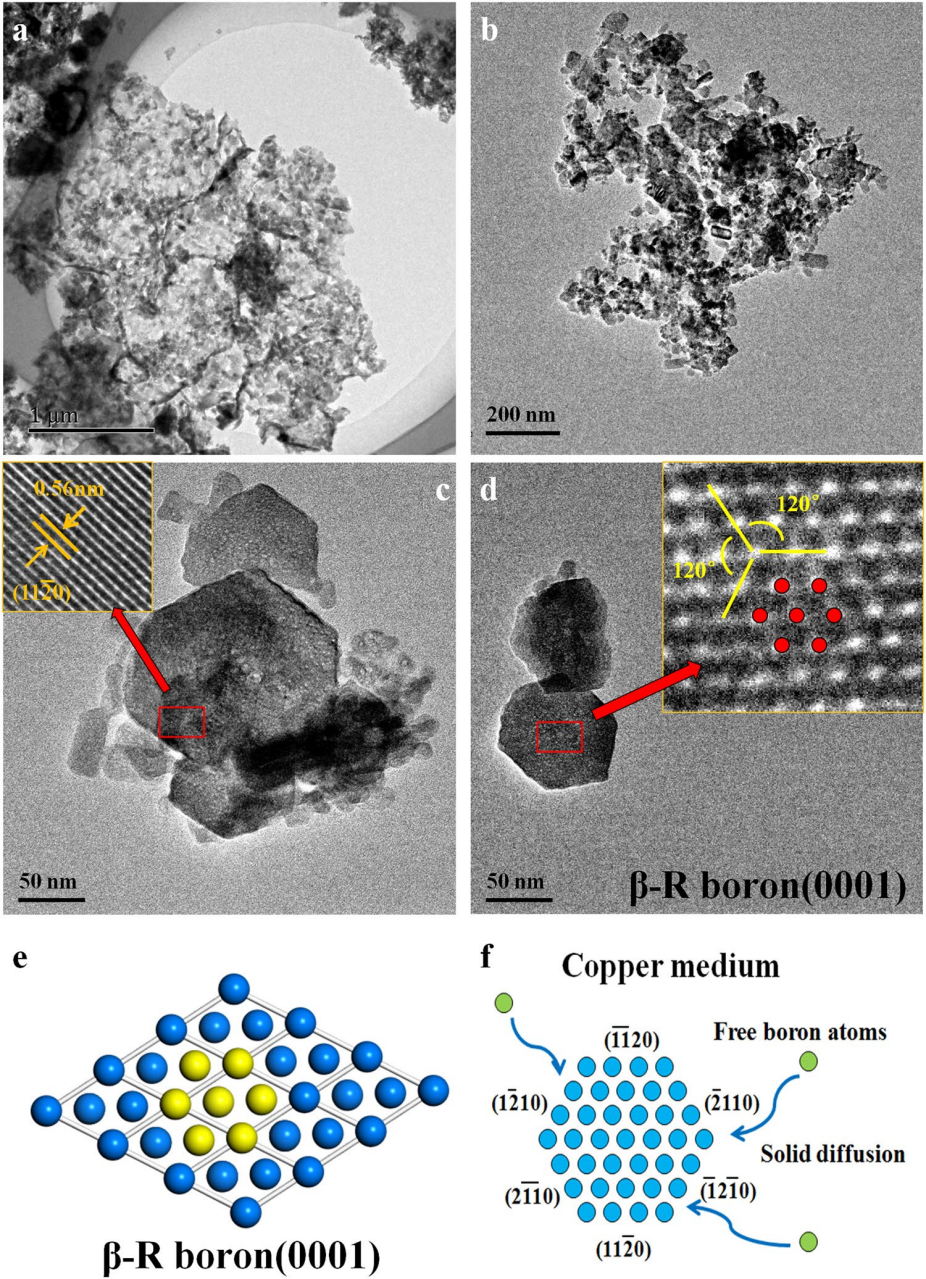
The characterization of hexagonal boron nanosheets. (**a**,**b**) TEM images of the nanosheets. Boron nanosheets gather together due to a large specific surface area; (**c**,**d**) TEM and HRTEM images of the hexagonal boron nanosheets. The crystal plane spacing of 0.56 nm with an angle of 120 degree indicates that the exposed plane is (0001) plane of β-rhombohedra boron; (**e**) the model of (0001) plane has been built by Material Studio and the yellow atoms corresponds to the red in (**d**) observed in HRTEM; (**e**) the schematic diagram of hexagonal boron nanosheets. Free boron atoms move to the edge of hexagon by solid diffusion and the nanosheets grow up continuely.

**Figure 5:**
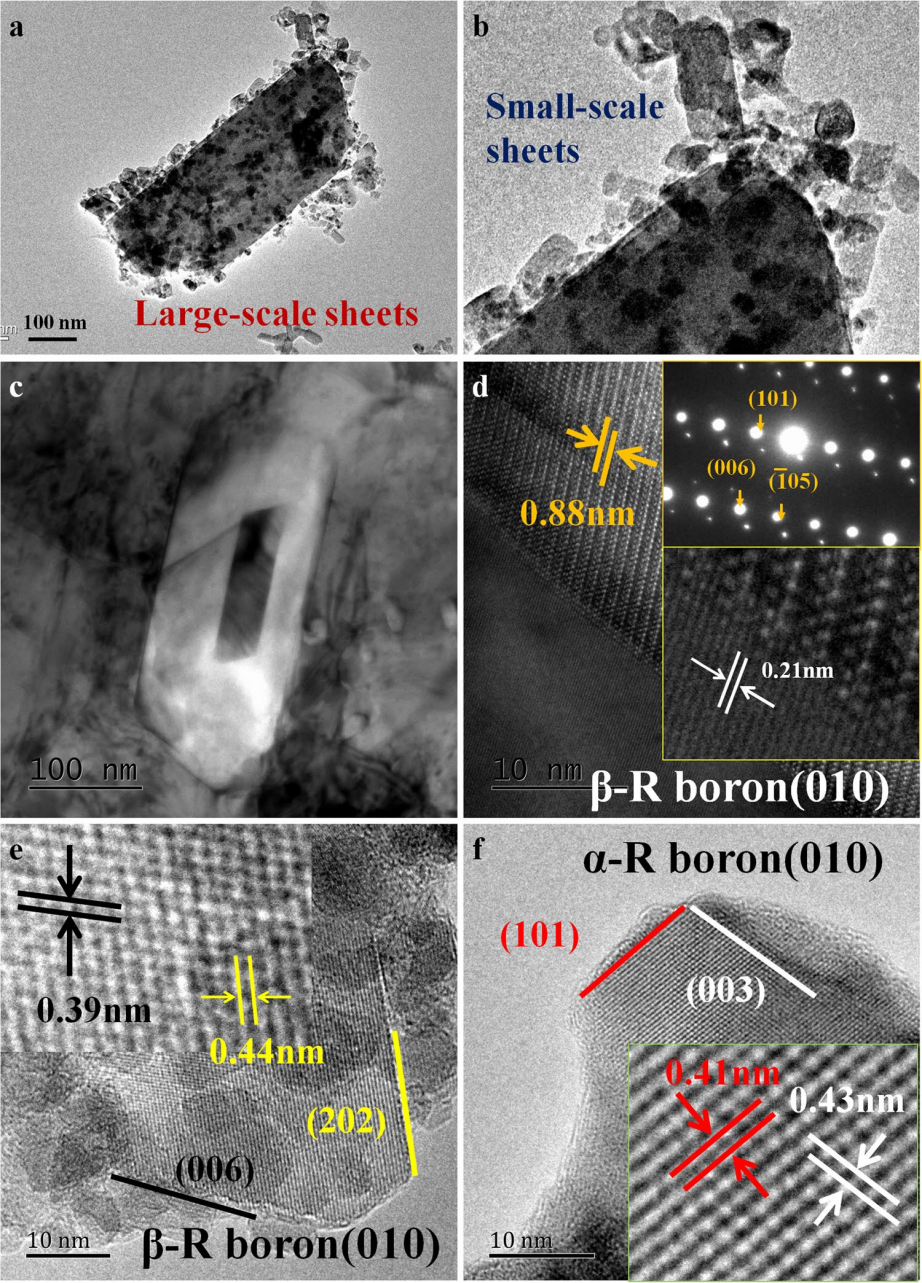
The characterization of rhombic boron nanosheets. (**a**,**b**) TEM images of rhombic nanosheets, with the size from several nanometers to several hundred nanometers; (**c**,**d**) TEM and HRTEM images of the hollow rhombic on copper matrix, showing that the exposed plane is (010) plane of β-rhombohedra boron and orientation relationship between copper and boron are investigated; (**e**) the HRTEM image of rhombic nanosheets, (202) and (101) are marked by yellow and black respectively; (**f**) the HRTEM image of another typical nanosheet, having the lattice characteristics of α-rhombohedra boron.

Figures 4 and 5 show the TEM images of boron nanosheets. Two basic shapes, hexagon and rhomboid, are observed clearly, which may suggest the different crystal faces of boron as well as two different growth mechanisms. Among them, the hexagonal boron nanosheets are shown in Fig. 4(c,d), the crystal lattice of boron also can be analyzed by the inserted image, so as to characterize the boron nanosheets in detail. The crystal plane spacing along one edge of the hexagon was measured at about 0.56 nm as labeled by orange lines in Fig. 4(c), consistent with that of β-rhombohedra boron (11–20) plane. To confirm this result, another hexagonal nanosheet is shown in Fig. 4(d), in which three planes with different directions are marked by yellow lines and the angles between the three planes are 120 degrees, implying that the planes corresponding to each side of the hexagon belong to (11–20), because in a hexagonal system, the crystallographic orientations of cylinders perpendicular to x-y axis are equivalent, being members of a same family of planes. The crystal lattice of hexagonal boron nanosheet is marked by red dots in the inserted image of Fig. 4(d) and the (0001) plane of β-rhombohedra boron has been also drawn by the software Material Studio 7.0, according to PDF31-0207(the unit cell parameters: a = 10.925 Å, b = 10.925 Å and c = 23.814 Å). In comparison, the plane of the hexagon boron nanosheets obtained in our experiment is indeed the (0001) plane of β-rhombohedra boron. Based on above analysis, the growth mechanism of hexagonal boron nanosheets, that the three planes corresponding to the six edges belong to the crystal plane family {11–20}, has been presented in Fig. 4(f).

In addition to hexagons, rhombic boron nanosheets, the sizes of which value from several nanometers to several hundred nanometers, have also been observed in large quantities as shown in Fig. 5(a,b). In our previous study^20^, boron nanotubes were prepared successfully in copper melt with a relatively high cooling speed, meanwhile the simulation told us that the nanotubes obtained in copper were quite unstable and have a great tendency to transform into regular polygons. Interestingly, a hollow rhomboid was noticed in boron/copper composite without extraction process and we regard it as an important evidence to judge its growth mechanism definitely. Figure 5(c,d) show this rhombic boron on copper matrix and the HRTEM images have been also analyzed. The X-ray diffraction result reveals that it exhibits a typical structural characteristic of β-rhombohedra boron and the two sides of the rhomboid are (101) plane and (006) plane. Besides, the two plane spacings aside the interface are also measured at about 0.88 nm in boron and 0.21 nm in copper matrix as labeled in Fig. 5(d), corresponding to β-R boron (101) plane and copper (111) plane, respectively, indicating that the orientation relationship between these two phases might play an important role in the preferred growth of boron. In consequence, it is speculated that the rhombic boron nanosheets are most likely to evolve from boron nanotubes, which have been broken via ball milling, under high temperature and pressure. Nevertheless, are the boron nanosheets extracted from copper consistent with those observed *in situ*? Fig. 5(e) shows a HRTEM image, revealing the lattice of rhombic boron. Here, the atomic layers inserted into the (101) planes, having been observed in Fig. 5(d) faintly, are clearly shown, for which the two plane spacings of two sides of the rhomboid are measured at 0.44 nm and 0.39 nm, consistent with the (202) plane and (006) plane of β-rhombohedra boron. In addition, we measured the angle between the two sides at 68 degrees, being equal with the angle calculated in the model of β-rhombohedra boron as well, which confirms our previous judgment of this crystal structure. However, it’s not the only structure. Figure 5(f) shows a different rhomboid, the angle between two sizes of which is measured at about 73 degrees and the plane spacings are either not same as that investigated above. According to the Raman spectra analyzed before, both β-rhombohedra and α-rhombohedra boron have been obtained in our experiment, so could it possess the characteristics of α-rhombohedra boron? The two planes corresponding to the sizes of rhomboid are marked by red and white solid lines in Fig. 5(f), the spacings of which are 0.41 nm and 0.43 nm, consistent with the (101) and (003) planes of α-rhombohedra boron, respectively, and the angle between them is about 71.5 degrees theoretically. It can be noticed that these two planes parallel with that of β-rhombohedra boron studied above. In fact, the structures of and α-rhombohedra boron are very similar, both belonging to R-3m (No. 166) space group. Therefore, the existence of α-rhombohedra boron nanosheets is definitely reasonable, considering the similarity between it and β-rhombohedra boron.

**Figure 6:**
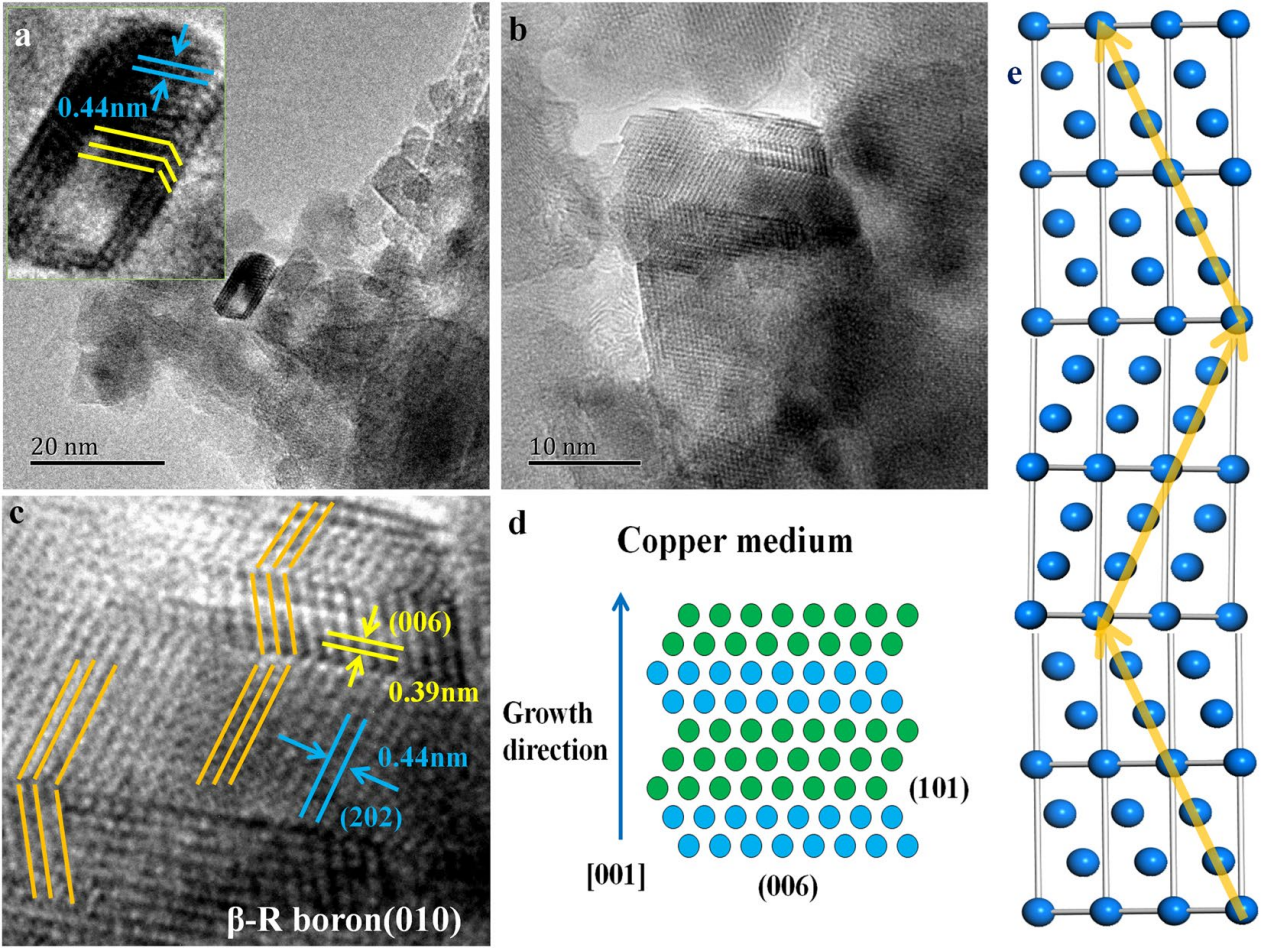
High density twins observed in β-rhombohedra boron nanosheets. (**a**,**b**) HRTEM images of twins noticed in rhombic nanosheets, the crystal spacing is measured at about 0.44 nm and the twins are labeled by yellow lines; (**c**) the magnified version image of (**b**), indicating that the twinning plane is (006) of β-rhombohedra boron; (**d**) the schematic diagram of twinning growth in β-rhombohedra boron nanosheets and the growth direction of twins is [001]; (**e**) the model of twins was built in Material Studio.

Surprisingly, twins are also observed in rhombic boron nanosheets with relatively small size as shown in Fig. 6(a,b). Although the twins existed in β-rhombohedra boron have been discussed in our previous studies^25^, it is still the first time to discover nanoscale twins like this in boron. Zhiyang Yu *et al*. have investigated the growth habit of boron-rich low-dimensional materials, revealing that two basic contact twinning, parallel twinning and cyclic twinning, could induce the growth of boron-rich materials^26^. Here we applied direct TEM observation to uncover the mechanism of nanotwins in boron. Figure 6(a) shows a small-scale sheet with about ten nanometers in length and there are two twin boundaries in the width direction, though the width values only five nanometers, just like three typical boron nanosheets are connected side by side. The crystal planes with a symmetrical relationship along the twin boundary have been marked by yellow solid lines and the spacing is about 0.44 nm, for which it is considered to be (202) plane according to previous analysis in Fig. 5(e). In order to confirm this speculation, a more clear twin image and the magnified version are shown in Fig. 6(b,c), respectively. The orange solid lines depicted the crystal planes with a symmetrical characteristic, the spacing of which is 0.44 nm, while the spacing of another plane parallel with twin boundary is about 0.39 nm, consistent with the (006) plane of β-rhombohedra boron. Distinctly, high density twins are formed during the crystallization process of rhombic boron nanosheets and the twinning plane is (006). The growth of the twins have been depicted in Fig. 6(d) briefly. When the boron nanosheet grows, there is a high tendency to form twins along the [001] direction. A slow cooling rate after HT and HP process may play an important role to the formation of twins, which could be considered as annealing twins. As shown in Fig. 6(e), the model of β-rhombohedra boron was built in MS and the (010) plane was cleft to express it vividly, in which the (202) plane were marked by orange solid lines, twins are represented as well. Ke Lu *et al*. reported a copper alloy with high density nanotwins, which possessed ultrahigh strengthen and electric conductivity, stimulating a great interest in studying twins or stacking faults^27^. Yongjun Tian also obtained a superhard boron nitride with a hardness even higher than diamond^28^. Certainly, it is valuable to study the high density twins on boron nanosheets, since the coherent twin boundaries could also be induced in materials to improve mechanical properties^29^.

In summary, boron nanosheets have been obtained in large quantities through a low cost method. The solid diffusion of boron at high temperature and pressure is the main approach. AFM images indicate that the thicknesses of boron sheets are usually less than 6 nm. Two structures, α-rhombohedra boron and β-rhombohedra boron, are detected by Raman spectrum. Elemental Al, Cu and O are dissolved in boron nanosheets through XPS and EDS. Hexagonal and rhombic sheets, corresponding to (0001) and (010) planes of boron respectively, are observed by HRTEM. Therein, copper plays a vital role in the growth of rhombic sheets. Moreover, the high density nanotwins provide a great potential for the application of boron nanosheets.

## Methods

**Figure 7:**
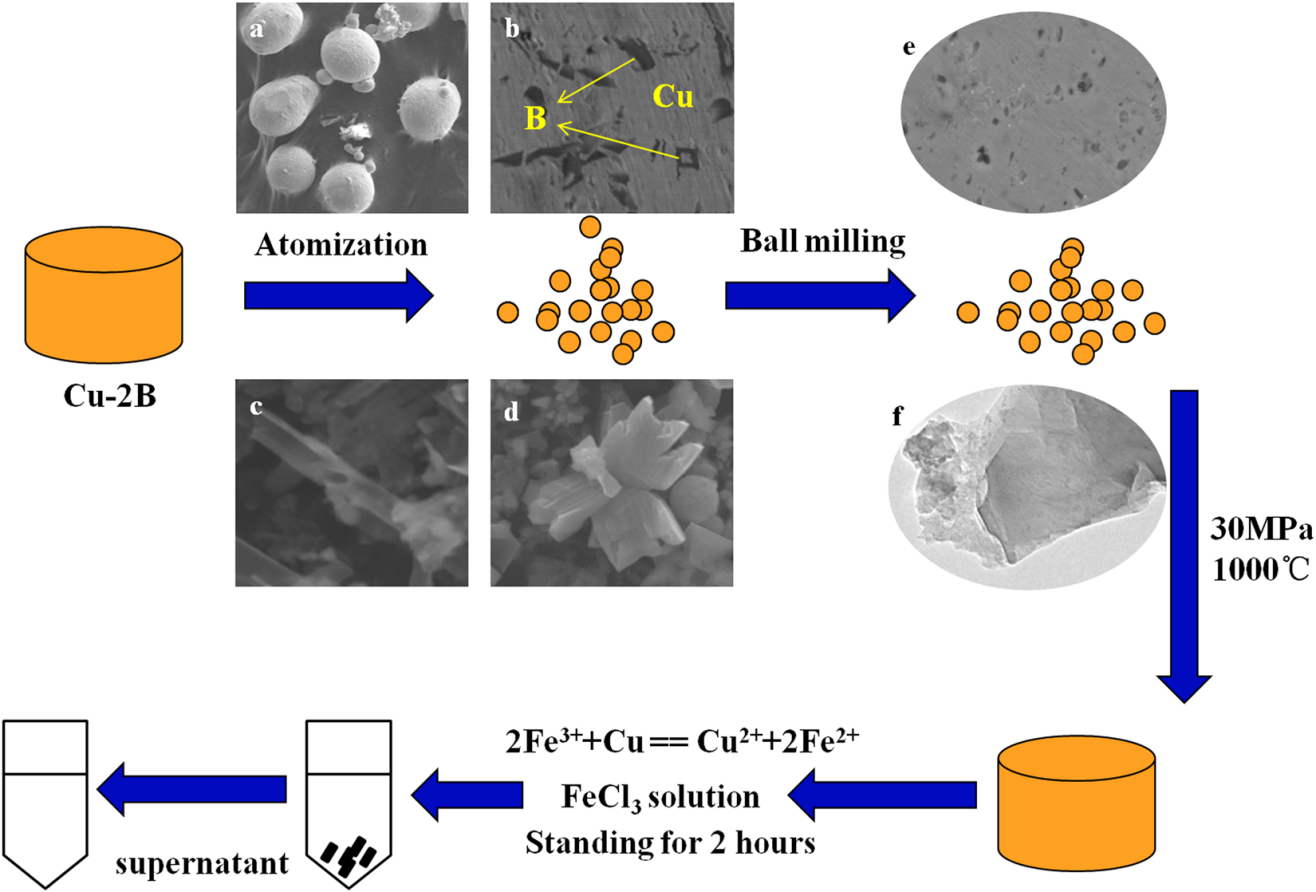
Preparation of boron nanosheets. Atomization and ball milling are the processes of mechanical exfoliation, by which bulk boron in copper are broken into pieces. High temperature and high pressure (HTHP) provide the energy for solid diffusion of boron atoms in copper medium. And then boron is extracted from copper using FeCl_3_ solution and boron nanosheets were suspended inside the supernatant after two hours standing.

In this work, Cu-B alloys prepared by a reduction reaction^19^ were atomized into powders, using pure Cu( > 99.9%), pure Al(>99.9%) and B_2_O_3_. 2 wt% boron content was selected in our experiments due to the eutectic point of Cu-B alloys, in which the low melting temperature facilitates atomization process. Figure 7(a–d,c,d) show the microstructures of atomized Cu-B powders and extractive from that respectively, corresponding to products after atomization process, the black phase in Fig. 7(b) is boron and the medium is copper. Two structures, tubes and bulks were observed by this step. The ball milling process, with 4:1 ball/powder ratio and 360r/min rotation speed, was applied to grind the Cu-B powders for 18 hours, by which the structure of eutectic boron could be destroyed, even reaching an amorphous state. Microstructures of milled powders are demonstrated in Fig. 7(e) and sheet structure could be obtained after extraction as shown in Fig. 7(f). Then the milled powders were compacted in graphite mould and recrystallization took place in a hot pressure sintering furnace under a vacuum condition (<10^−5^ Pa), accompanied by 1000 °C and 30 MPa. High temperature leads to the crystallization of boron, while high pressure prevents the excessive growth of boron, by which solid copper restricts the growth of boron, resulting in the formation of boron nanosheets. Finally, FeCl_3_ solution was used to extract boron from copper by the reaction, 2Fe^3+^ + Cu = = Cu^2+^ + 2Fe^2+^. Herein, boron nanosheets were suspended inside the supernatant after two hours standing and could be extracted easily by a straw. Figure 7 depicts the entire process vividly.

Microstructures characteristic was performed utilizing High-Resolution Transmission Electron Microscope (HRTEM, JEM-2100, Japan), field emission scanning electron microscope equipped with EDS (FESEM, SU-70 SEM, Japan), Scanning Probe Microscope (SPM, Dimension Icon, America) and FEI Titan G2 TEM(ETEM, German). Phase identification was characterized by inVia Raman Microscope with the excitation wavelength of 632.8 nm. The chemical composition of the boron nanosheets was analyzed by X-rays Photoelectron Spectroscopy (Axis Supra).
